# Consequences of Peri-Implant Bone Loss in the Occlusal Load Transfer to the Supporting Bone in terms of Magnitude of Stress, Strain, and Stress Distribution: A Finite Element Analysis

**DOI:** 10.1155/2021/3087071

**Published:** 2021-09-01

**Authors:** Esteban Pérez-Pevida, David Chávarri-Prado, Markel Diéguez-Pereira, Alejandro Estrada-Martínez, Oier Montalbán-Vadillo, Antonio Jiménez-Garrudo

**Affiliations:** ^1^Department of Surgery, Faculty of Medicine, University of Salamanca, Salamanca, Spain; ^2^Faculty of Dentistry, Miguel de Cervantes European University, Valladolid, Spain; ^3^Department of Surgery and Medical-Surgical Specialties, Faculty of Medicine, University of Oviedo, Oviedo, Spain

## Abstract

**Methods:**

Three models of a single internal connection bone level-type implant inserted into a posterior mandible bone section were constructed using a 3D finite element software: one control model without marginal bone loss and two test models, both with a circumferential peri-implant bone defect, one with a 3 mm high defect and the other one 6 mm high. A 150 N static load was tested on the central fossa at 6° relative to the axial axis of the implant.

**Results:**

The results showed differences in the magnitude of strain and stress transferred to the bone between models, being the higher strain found in the trabecular bone around the implant with greater marginal bone loss. Stress distribution differed between models, being concentrated at the cortical bone in the control model and at the trabecular bone in the test models.

**Conclusion:**

Marginal bone loss around dental implants under occlusal loading influences the magnitude and distribution of the stress transferred and the deformation of peri-implant bone, being higher as the bone loss increases.

## 1. Introduction

Dental implants have become the most widely used and predictable way to treat edentulism, with high rates of success and long-term survival [1]. Nevertheless, treatment with dental implants is not without its limitations and complications. Peri-implantitis, defined as the infection of the tissue surrounding a dental implant characterized by inflammation of the peri-implant connective tissue and by a progressive loss of supporting bone in an accelerated and nonlinear pattern, is among the most frequent complications [2, 3].

Nowadays, although periodontal and peri-implant diseases are some of the most studied topic in dentistry [3], the etiology of peri-implant diseases remains controversial. Peri-implantitis has traditionally been considered a bacterial infection, similar to that assumed for periodontal disease. Accordingly, evidence regarding the role of certain periodontopathogenic bacteria in the infectious etiology of peri-implantitis, including *Porphyromonas gingivalis*, *Treponema denticola*, and *Tannerella forsythia*, is moderate, whereas evidence for other types of bacteria, including *Prevotella intermedia* and *Campylobacter rectus*, is mild [4].

Other trends have advocated the primary inflammatory etiology of periodontal disease over bacterial infection alone. Accordingly, Hajishengallis and Lamont argued that commensal microbial complexes transition into pathogenic complexes as a result of uncontrolled inflammation in the periodontal or peri-implant area. Communication between bacterial species leads to synergy between metabolically compatible organisms that have acquired functional specialization. The researchers also argued that key pathogens, such as *Porphyromonas gingivalis*, even in low abundance, subvert the nonspecific immune system of the host, causing sustained inflammation, evading phagocytosis, and suppressing its bactericidal capacity, both at the cellular and complement levels, thereby leading to bacterial dysbiosis, which increases the virulence of the entire bacterial complex [5–7]. Although these studies refer to the etiology of periodontal diseases, they should be taken into account in the assessment of the etiology of peri-implant diseases due to the degree of similarity between both diseases.

Therefore, the bacterial etiology of classical periodontopathogens is currently under discussion, further highlighting the role of an inflammatory reaction. Inflammation, through the activation of key pathogens, leads to this dysbiosis of the bacterial microbiota, in turn destroying the host tissue.

Numerous factors may affect the onset and progression of peri-implantitis, including the patient, surgical technique, implant, or implant-supported prosthesis applied [8–12]. Other factors apparently also contribute to marginal bone loss, despite a lack of strong evidence supporting their role. Accordingly, an occlusal overload affects peri-implant marginal loss. However, because of difficulties in conducting clinical trials evaluating this variable, only *in vitro* assays and animal model studies comparing this effect are currently available [13–17]. To assess the effect of an occlusal overload on peri-implant bone, the biomechanical behavior of the prosthesis-implant-bone system must be understood.

Wolff's law states that bones undergo remodeling according to the forces to which they are subjected during their function, modifying their internal and external architecture and in turn altering their shape and density [18, 19]. Mechanically, bones behave similarly to any other material, undergoing strain when subjected to a load. Therefore, all bones are capable of withstanding some strain, which causes microfractures and leads to bone loss. This microstrain clinically translates into micromovements of the tooth or implant; micromovements of greater than 150 *μ*m are poorly tolerated by the bone-implant system and may result in the failure of the osseointegration process [20]. Nevertheless, there are no studies assessing the micromovement required in order to lose implant osseointegration once achieved, although the available evidence suggests that forces that trigger micromovements exceeding the elastic limit of the bone may cause the loss of bone-implant union.

Several studies have shown the influence of various factors on the way in which stress transfer occurs to the peri-implant marginal bone, as a result of the application of a functional or parafunctional load, such as the macroscopic design and surface treatment of the implant, the type of load, the quantity and quality of the peri-implant bone, and the properties of the prosthesis and implant material [21, 22].

However, there is limited evidence of the effect of peri-implantitis on a load transfer to the support bone, that is, how the amount of marginal bone loss around the dental implant affects the biomechanical behavior of the prosthesis-implant-bone system.

The objective of this finite element analysis is to assess the effect of the amount of marginal bone loss on the load transfer to the peri-implant bone in terms of the magnitude and distribution of stress and strain.

The null hypothesis of this work is that the loss of peri-implant marginal bone does not produce changes in the amount and distribution of stress transferred to the support site.

## 2. Materials and Methods

### 2.1. Finite Element Model Design

A type-3 edentulous posterior mandible bone section was designed according to the classification by Lekholm and Zarb [23]. The bone surrounding the implant was 23 mm high and 12 mm wide with a 1 mm thick cortical bone section, with the remaining section consisting of trabecular bone.

In the macroscopic design of the threaded implant, a standard Ti-6Al-4V alloy internal connection implant with a 2.8 mm smooth neck section (Straumann Standard, Institute Straumann AG, Basel, Switzerland), 10 mm in length, 4.1 mm in body diameter, and 4.8 mm in platform diameter, was used as a reference. The implant body is located with the treated surface below the bone crest in the cortical bone, leaving a smooth neck outside the bone and thus promoting the ideal placement of an implant with such characteristics. A corresponding platform with a 4.8 mm diameter and a 5.5 mm high titanium screw-retained abutment was modeled (RN synOcta, Institute Straumann AG, Basel Switzerland).

An 8 mm high, 10.6 mm wide, and 3 mm thick Cr-Co alloy metal-ceramic crown (1 mm metal alloy and 1 to 2 mm ceramic coating) with feldspathic ceramic coating cemented on a titanium abutment was modelled.

**Figure 1:**
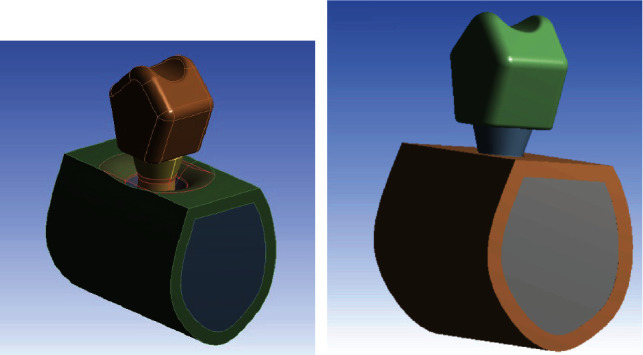
Finite element model with circumferential bone defect (a) and without marginal bone loss (b).

Three 3D finite element models were designed to evaluate the magnitude and distribution of peri-implant bone stress, namely, a control model without marginal bone loss, and test models 1 and 2 with a 3 mm and 6 mm circumferential bone defect around the implant, respectively (Figure 1).

### 2.2. Properties of the Materials and Interface Conditions

The properties of all materials used in the finite element models were extracted from the literature and are outlined in Table 1 [21]. All materials used in these models are considered linearly elastic, homogenous, and isotropic. Ideal (100%) osseointegration in the interface between the bone and implant was assumed in this study. The cement layer between the crown and abutment was omitted, considering an exact passive fit and an effective bonding between both components.

### 2.3. Loading and Boundary Conditions

The models were developed and analyzed using the Ansys 11.0 3D finite element modeling software (Ansys, Swanson Analysis System, Canonsburg, PA, USA).

Under all assumptions, a load of 150 N was applied to the central occlusal fossa of the crown in the vestibule-lingual direction and with a 6° angle with respect to the axial axis of the implant, thus promoting the physiological loading conditions of a mandibular premolar-molar sector [24].

Numerical von Mises stress and strain and stress data were collected from all finite element models and encoded into colorimetric scales to more easily compare the differences between study models.

The finite element model of the control used in this study consisted 33,268 elements and 45,517 nodes, test model 1 consisted 706,329 elements and 1,073,794 nodes, and test model 2 consisted 752,945 elements and 1,084,077 nodes.

## 3. Results

The results of the finite element analysis of the three study models showed the magnitudes of the von Mises stress and strain of the cortical bone, trabecular bone, and implant, as well as the distribution of the stress transferred to the prosthesis-implant-bone complex.

The results of the maximum von Mises stress transferred to the cortical bone, trabecular bone, and implant are outlined in Table 2. In cortical bone, the highest maximum tension transfer occurred in the control model without marginal bone loss at 16.945 MPa, whereas the lowest maximum stress transfer occurred in the test model with 6 mm of marginal bone loss at 5.8849 MPa. The opposite results were found in the trabecular bone in which the highest maximum stress transfer occurred in the test model with 6 mm of marginal bone loss at 9.995 MPa, whereas the lowest maximum stress occurred in the control model without peri-implant loss. The implant subjected to the highest stress was the control model without bone loss (91.23 MPa), whereas the implants of both models with marginal bone loss were under a lower stress, with 53.678 MPa for the model with 3 mm of loss and 56.861 in the model with 6 mm of loss.

The strain, expressed in microns (*μ*m), found in the cortical and trabecular bone and in the implants of the three study models, is outlined in Table 3. In cortical bone, the highest strain was observed in the control model without bone loss (6.251 *μ*m) and the lowest value appeared in the model with 6 mm of loss (3.408 *μ*m). The opposite results were found in trabecular bone, wherein the highest strain was assessed in the model with 6 mm of loss (14.899 *μ*m) and the lowest in the control model without bone loss (6.055 *μ*m). The maximum strain values were significantly higher in trabecular bone than in cortical bone for the test models with bone loss. The implant showed results similar to those of trabecular bone, wherein the highest strain was found in the implant of the model with 6 mm of loss (36.392 *μ*m) and the lowest in the implant of the control model without bone loss (8.314 *μ*m).

**Figure 2:**
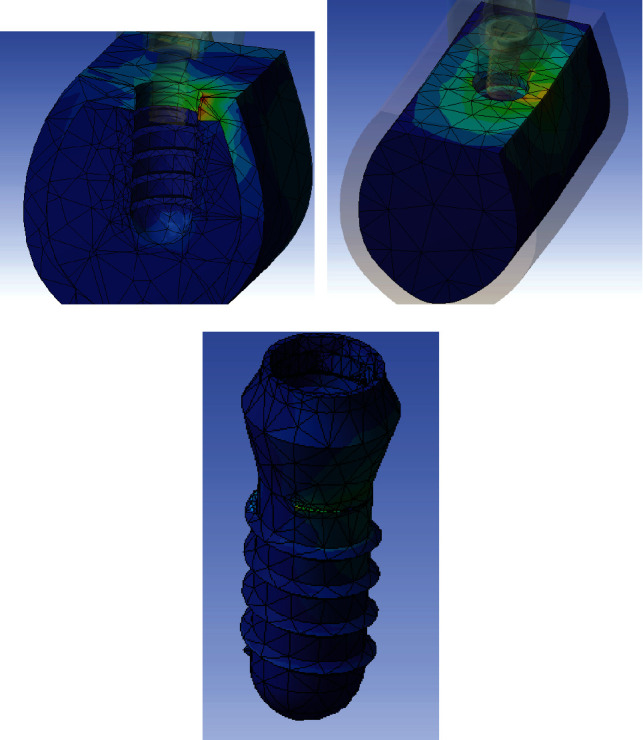
Stress distribution in cortical bone (a), trabecular bone (b), and implant (c) for the control model without marginal bone loss.

**Figure 3:**
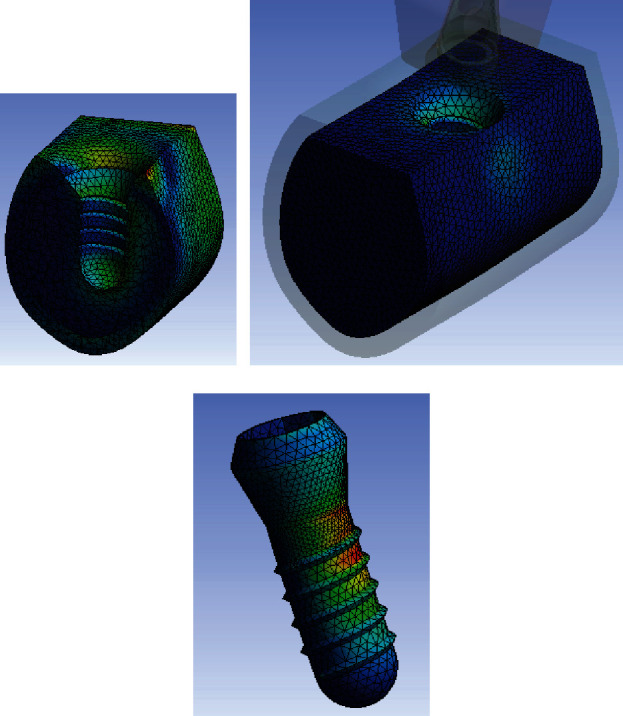
Stress distribution in cortical bone (a), trabecular bone (b), and implant (c) for the test model with a 3 mm high circumferential defect.

**Figure 4:**
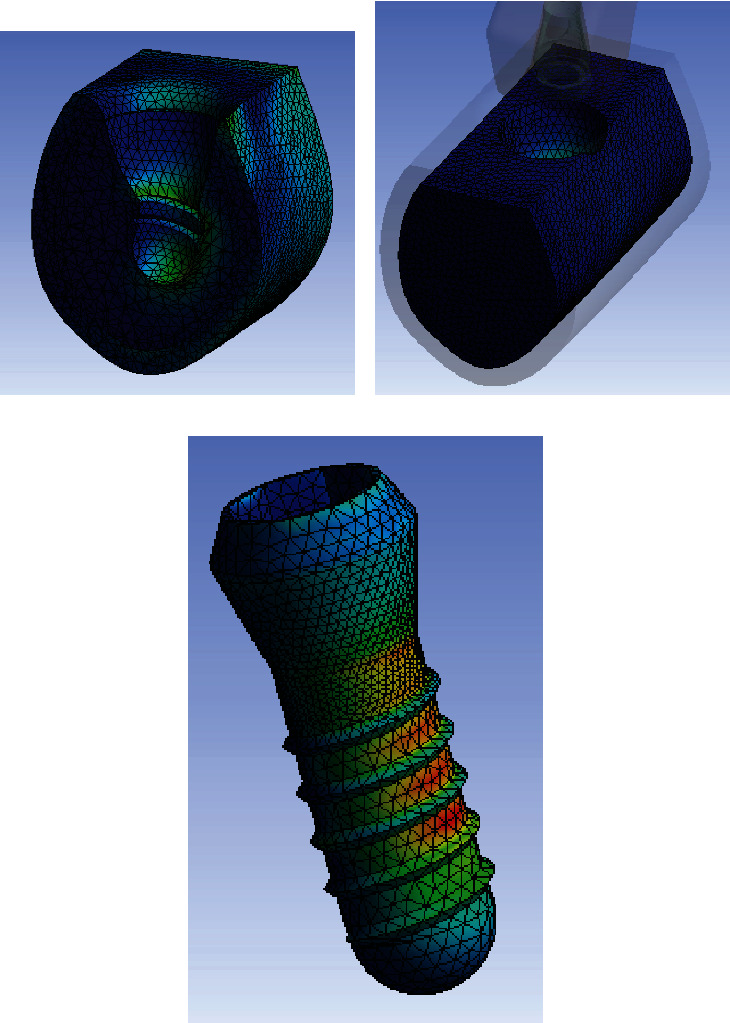
Stress distribution in cortical bone (a), trabecular bone (b), and implant (c) for the test model with a 6 mm high circumferential defect.

The analysis of the color charts of the study models showed marked differences in the distribution of the strain transferred to the prosthesis-implant-bone system (Figures 2–4). In the three study models, most of the peri-implant bone stress was located in the coronal bone in contact with the implant. Accordingly, in the control model without bone loss, the stress was primarily located in the peri-implant cortical bone, and to a lesser extent, in the bone surrounding the apex of the implant. In both test models with marginal bone loss, the stress distribution changed because the cortical bone was not in contact with the implant; therefore, the stress was essentially transferred in the coronal area of the trabecular bone in contact with the implant, transferring more apically in the test model with the greatest bone loss around the implant. In the three models, the stress was primarily transferred to the peri-implant bone on the side of the direction of the applied load vector. In this case, the vector has a buccolingual direction. Therefore, the stress was mostly distributed in the lingual sector of the bone around the implant. Some stress was also transferred to the bone adjacent to the apex of the implant, which responds to the axial component of the load applied to the model.

## 4. Discussion

This study uses a 3D finite element analysis to compare the magnitude and distribution of the stress and strain of the peri-implant bone and of the implant itself depending on the existence of marginal bone loss and its magnitude.

Based on these findings, the null hypothesis of this study is rejected because significant differences are found in the stress transfer depending on the presence of marginal bone loss around the implant. However, these results must be interpreted with caution because the validity of the finite element analysis of the stress depends on the degree to which the properties of the materials, their geometry, the applied load, and the conditions of the interface adjust to reality [25]. During this test, the structures simulated in the models were assumed to be homogeneous, isotropic, and linearly elastic, although this does not correspond to reality, particularly regarding bone. However, this assumption was required to simplify the model to complete the analysis, similar to numerous finite element tests evaluating the variations in stress in models of single implants [26–29].

As in most biomechanical finite element analyses, in this study, the trabecular and cortical bones have the same mechanical properties throughout the model because we assume a preestablished osseointegration. In this regard, studies have provided different properties of bone in close contact with an implant and the rest of the modeled bone, simulating a transitional bone undergoing healing during the osseointegration process [30].

The occlusal load used in the present analysis was 150 N at 6° with respect to the axial axis of the implant, thus simulating the average values collected in a patient with a dental implant, which are considered a physiological occlusal force similar to the masticatory forces [24]. However, the force tested in this analysis is essentially static, corresponding to the parafunctional force typical of centric bruxism, whereas the masticatory forces are fundamentally dynamic. The type of load and the elastic properties of the modeled materials may affect the biomechanical results, and these limitations must be considered when evaluating the findings.

After applying the load, the three studied models follow the same pattern of stress transfer to the peri-implant bone. This stress is primarily transferred to the coronal bone in contact with the implant, in line with the principle of a composite beam analysis, which stipulates that, when two materials with different elastic modulus (bone and implant in this test) are brought into contact and one of them is subjected to a load, the greatest stress is transferred to the first point of contact between the two materials, that is, to the most coronal bone in contact with the implant [31]. Accordingly, in the control model without bone loss, the stress is mostly transferred to the cortical bone in contact with the implant; conversely, in the test models with a bone defect around the implant, the stress is primarily transferred to the trabecular bone because this marginal bone loss coincides with the cortical bone loss.

The design of this peri-implantitis model, with cortical bone loss, is in line with other finite element analysis [4], but some studies have modeled cortical bone in peri-implant defects [26, 32, 33]. In this regard, this study assumes that marginal bone loss results from an active peri-implant disease, and therefore, based on histological studies, from the osteoclastic activity of the cortical bone characterized by the presence of Howship's lacunae with numerous resident osteoclasts [34–36]. Nevertheless, some studies have shown partial bone corticalization in peri-implant defects preestablished by a biomechanical reinforcement of the residual trabecular bone [37].

The analysis of the results of the magnitude of stress transferred to the bone in the three models shows that the maximum stress is transferred to the cortical bone of the control model without bone loss, similar to numerous finite element analyses of single implants without bone loss [21, 27–29]. However, when evaluating the stress transferred to the trabecular bone, we observed that the maximum stress increases with the increase in the marginal bone loss. In addition, the highest value was found in the test model with a 6 mm defect around the implant. These data match those found in several finite element analyses assessing the effect of the peri-implant bone resorption on the load transfer to the support ground, with a directly proportional relationship between the increased bone loss and increased stress transferred to the bone [32, 33, 38, 39].

However, the maximum value of stress transferred to the cortical bone in the control model without bone loss (16.945 MPa) is higher than the maximum value of stress transferred to the trabecular bone in the test model with 6 mm of bone loss (9.995 MPa). However, the importance of these results lies in Hooke's law, according to which *σ* = *E* · *ε*; that is, when applying stress (*σ*) to a material with a specific modulus of elasticity or Young's modulus (*E*), this body experiences strain (*ε*), which is directly proportional to the applied stress [40]. That is, when applying the same occlusal load, the increased stress transferred to the trabecular bone is more decisive because Young's modulus of the trabecular bone is lower than that of the cortical bone, and therefore, the former tends to suffer greater strain than the latter.

These data match the bone strain values determined during this trial, wherein the highest strain was found in the trabecular bone of the test model with the greatest bone loss. In other words, although the maximum value of stress was found in the cortical bone of the control model, the maximum strain was assessed in the trabecular bone of the model with a 6 mm defect around the implant. These strain data corroborate the findings of Akca and Cehreli, which is the only finite element analysis found evaluating the peri-implant bone strain in models with a different progression of marginal bone loss. In this analysis of models with marginal bone loss ranging from 0 to 2 mm with 0.25 mm increments, bone strain is directly proportional to the height of the peri-implant defect [38].

In this regard, Frost proposed a criterion for bone remodeling based on the magnitude of the internal stress that the bone experiences while performing its function; that is, the bone is able to withstand strain up to a specific threshold from which microfractures will occur, which in turn leads to bone loss [18]. More recently, Wiskott and Belser established a set of bone remodeling categories, from disuse atrophy to fracture, through a series of bone adaptation windows, depending on the strain experienced by the bone. In this sense, the bone which suffers less than 100 microns of deformation (*με*) may be likely to suffer from bone disuse atrophy and, in the other hand, may be at risk of fracture under more than 25,000*με*. In between these two extremes, three ranges can be defined, a normal load with bone homeostasis (100 − 2000*με*), mild overload with an increase of bone mass (2000 − 4000*με*), and pathologic overload with an irreversible bone damage (4000 − 2500*με*) [41].

Extrapolating the data collected in this trial to a clinical setting, all bone strain values assessed in this test are within the range of physiological bone adaptation without a risk of anabolic bone reaction owing to excess strain. However, the load applied to the three models was a static load of 150 N, compatible with a functional physiological magnitude; thus, the strain values should be evaluated when simulating a parafunctional occlusion or occlusal alteration with a considerable increase in the magnitude of the applied load. Therefore, if we apply a greater force simulating a parafunctional pattern or an occlusal alteration, such as a premature contact or interference, the presence of higher strain values close to the pathological overload limit described by Wiskott may be feasible, particularly in implants with great marginal bone loss. In this sense, an exhaustive clinical management of occlusal factors should be recommended when marginal bone loss occurs around dental implants in order to minimize the load transferred to peri-implant bone, such as occlusal adjustments or modifications of the rehabilitation materials.

On the other hand, both models with peri-implant bone loss show higher values of deformation of the trabecular bone which has lower elastic limit compared to cortical bone. This result clinically may lead to a faster and higher progression of peri-implant disease due to the poor tolerance of trabecular bone to higher values of deformation, which could result in a progressive bone loss worsening the stage of the disease.

Finally, the results obtained in this study may have applications in the diagnosis of peri-implant bone loss using less invasive methods than traditional ones, such as resonance frequency analysis (RFA). In this sense, if marginal bone loss leads to a worse biomechanical behavior of bone-implant complex with higher bone deformation and implant micromovement, it could be diagnosed using RFA devices with a decrease in implant stability quotient (ISQ) values. In this respect, several studies have shown the influence of peri-implant bone defects on ISQ values using RFA devices, suggesting it a potential use in the diagnosis of peri-implant disease [42, 43].

Given the limitations of extrapolating results to clinical practice, and the fact that it is impossible to reproduce oral physiological and anatomical conditions exactly in finite elements analysis, the present results should be treated with caution.

## 5. Conclusions

According to the findings, and within the limitations of this *in vitro* study, we can deduce the following:

1. When an implant with marginal bone loss is subjected to a functional load, the trabecular bone tends to receive a higher stress and therefore a higher strain as this loss increases
2. Increased bone strain may increase the risk of pathological overload, which in the presence of a parafunction or unfavorable biomechanical situation may exacerbate the anabolic bone reaction
3. The results from this study reject the initial null hypothesis according to which the bone around an implant with marginal bone loss subjected to a functional load will tend to experience similar stress and therefore similar strain, regardless of the amount of bone loss, in comparison with the bone around an implant without peri-implantitis.

## Figures and Tables

**Table 1:** Mechanical properties of materials and fixtures.

Material	Component	Young's modulus (GPa)	Poisson's coefficient
Cortical bone		15	0.30

Trabecular bone		1	0.25

Ti-6Al-4V alloy	Abutment and screw	107.2	0.30
	Dental implant	110	0.35

Cr-Co alloy	Crown structure	218	0.33

Feldspathic ceramic	Crown veneering	65	0.25

**Table 2:** Maximum von Mises stresses (MPa) in cortical and trabecular bones and implants for all the models evaluated.

Model	Cortical bone	Trabecular bone	Implant
3 mm defect	5.934 MPa	8.109 MPa	53.678 MPa
6 mm defect	5.884 MPa	9.995 MPa	56.861 MPa
Control	16.945 MPa	2.038 MPa	91.23 MPa

**Table 3:** Maximum and minimum deformations (*μ*m) in cortical and trabecular bone and in implants for all the models evaluated.

Model		Cortical bone	Trabecular bone	Implant
3 mm defect	Min	0 *μ*m	0 *μ*m	5.691 *μ*m
	Max	3.4866 *μ*m	10.553 *μ*m	18.06 *μ*m

6 mm defect	Min	0 *μ*m	0 *μ*m	5.563 *μ*m
	Max	3.408 *μ*m	14.899 *μ*m	36.392 *μ*m

Control	Min	0 *μ*m	0 *μ*m	4.500 *μ*m
	Max	6.251 *μ*m	6.055 *μ*m	8.314 *μ*m

## Data Availability

The pictures of data used to support the findings of this study are included within the article. The entered sheet data of this study are available from the corresponding author upon request.
